# White Matter Magnetic Resonance Diffusion Measures in Multiple Sclerosis with Overactive Bladder †

**DOI:** 10.3390/brainsci14100975

**Published:** 2024-09-27

**Authors:** Xixi Yang, Martina D. Liechti, Baris Kanber, Carole H. Sudre, Gloria Castellazzi, Jiaying Zhang, Marios C. Yiannakas, Gwen Gonzales, Ferran Prados, Ahmed T. Toosy, Claudia A. M. Gandini Wheeler-Kingshott, Jalesh N. Panicker

**Affiliations:** 1 Department of Neurology, Xuan Wu Hospital of Capital Medical University, Beijing 100053, China; 2 Department of Brain Repair and Rehabilitation, Faculty of Brain Sciences, Queen Square Institute of Neurology, University College London, London WC1E 6BT, UK; martina.liechti@balgrist.ch (M.D.L.); j.panicker@ucl.ac.uk (J.N.P.); 3 Department of Uro-Neurology, The National Hospital for Neurology and Neurosurgery, London WC1N 3BG, UK; gwen.gonzales@uclh.nhs.uk; 4 NMR Research Unit, Queen Square MS Centre, Department of Neuroinflammation, UCL Institute of Neurology, University College London, London WC1E 6BT, UK; b.kanber@ucl.ac.uk (B.K.); gloria.castellazzi@unipv.it (G.C.); m.yiannakas@ucl.ac.uk (M.C.Y.); f.carrasco@ucl.ac.uk (F.P.); a.toosy@ucl.ac.uk (A.T.T.); c.wheeler-kingshott@ucl.ac.uk (C.A.M.G.W.-K.); 5 Department of Neuro-Urology, Balgrist University Hospital, University of Zürich, 8006 Zürich, Switzerland; 6 Centre for Medical Image Computing (CMIC), Department of Medical Physics and Biomedical Engineering, University College London, London WC1E 6BT, UK; c.sudre@ucl.ac.uk; 7 School of Biomedical Engineering and Imaging Sciences, King’s College London, London SE1 7EH, UK; 8 Dementia Research Centre, Institute of Neurology, University College London, London WC1E 6BT, UK; 9 Department of Electrical, Computer and Biomedical Engineering, University of Pavia, 27100 Pavia, Italy; 10 School of Artificial Intelligence, Beijing University of Post and Communications, Beijing 100876, China; zjyei2006@gmail.com; 11 Department of Computer Science and Centre for Medical Image Computing, University College London, London WC1E 6BT, UK; 12 e-Health Centre, Universitat Oberta de Catalunya, 08018 Barcelona, Spain; 13 Department of Brain and Behavioral Sciences, University of Pavia, 27100 Pavia, Italy; 14 Digital Neuroscience Centre, IRCCS Mondino Foundation, 27100 Pavia, Italy

**Keywords:** multiple sclerosis (MS), overactive bladder (OAB) symptoms, white matter (WM), diffusion tensor imaging (DTI), tract-based spatial statistics (TBSS)

## Abstract

Background: Lower urinary tract (LUT) symptoms are reported in more than 80% of patients with multiple sclerosis (MS), most commonly an overactive bladder (OAB). The relationship between brain white matter (WM) changes in MS and OAB symptoms is poorly understood. Objectives: We aim to evaluate (i) microstructural WM differences across MS patients (pwMS) with OAB symptoms, patients without LUT symptoms, and healthy subjects using diffusion tensor imaging (DTI), and (ii) associations between clinical OAB symptom scores and DTI indices. Methods: Twenty-nine female pwMS [mean age (SD) 43.3 years (9.4)], including seventeen with OAB [mean age (SD) 46.1 years (8.6)] and nine without LUT symptoms [mean age (SD) 37.5 years (8.9)], and fourteen healthy controls (HCs) [mean age (SD) 48.5 years (20)] were scanned in a 3T MRI with a DTI protocol. Additionally, clinical scans were performed for WM lesion segmentation. Group differences in fractional anisotropy (FA) were evaluated using tract-based spatial statistics. The Urinary Symptom Profile questionnaire assessed OAB severity. Results: A statistically significant reduction in FA (*p* = 0.004) was identified in microstructural WM in pwMS, compared with HCs. An inverse correlation was found between FA in frontal and parietal WM lobes and OAB scores (*p* = 0.021) in pwMS. Areas of lower FA, although this did not reach statistical significance, were found in both frontal lobes and the rest of the non-dominant hemisphere in pwMS with OAB compared with pwMS without LUT symptoms (*p* = 0.072). Conclusions: This study identified that lesions affecting different WM tracts in MS can result in OAB symptoms and demonstrated the role of the WM in the neural control of LUT functions. By using DTI, the association between OAB symptom severity and WM changes were identified, adding knowledge to the current LUT working model. As MS is predominantly a WM disease, these findings suggest that regional WM involvement, including of the anterior corona radiata, anterior thalamic radiation, superior longitudinal fasciculus, and superior frontal-occipital fasciculus and a non-dominant prevalence in WM, can result in OAB symptoms. OAB symptoms in MS correlate with anisotropy changes in different white matter tracts as demonstrated by DTI. Structural impairment in WM tracts plays an important role in LUT symptoms in MS.

## 1. Introduction

Lower urinary tract (LUT) symptoms are reported in more than 80% of multiple sclerosis (MS) patients (pwMS), significantly impacting quality of life [1,2]. The commonest LUT symptoms reported are storage or overactive bladder symptoms (OAB; defined as urinary urgency, with or without urgency urinary incontinence and usually associated with daytime urinary frequency and nocturia) [3] and voiding symptoms (including slow and/or intermittent stream, splitting or spraying, hesitancy, straining, and terminal dribble) [4,5]. The pattern of LUT symptoms is influenced by the site of lesions in the central nervous system [6].

In recent years, magnetic resonance imaging (MRI), in particular functional MRI (fMRI), has provided considerable insight into the central neural control of LUT functions [6,7]. There are a number of cortical and subcortical regions identified that consistently show changes in neural activity during bladder-specific tasks [8,9]. Insight has been gained into the role of these different regions such as the thalamus, insula, anterior cingulate cortex, and medial and lateral prefrontal cortex (mPFC and lPFC) during bladder filling and emptying, and distinct patterns of activation and deactivation are seen in cohorts of healthy subjects and in patients with idiopathic OAB [10]. These findings have led to a network model, which proposes cerebral circuits for perception of bladder fullness and urgency [8]. In MS, activation and deactivation have been seen in cortical and subcortical grey matter (GM) regions following a bladder infusion task [9,11]. These studies focus on GM changes; however, MS is overwhelmingly a white matter (WM) disease and the nature of the relationship between WM lesions and LUT symptoms is unknown. Studies using MRI showed more WM changes in MS, compared with healthy people, not only due to the WM lesions but also in the normal-appearing WM [12]. Regarding specific functions, microstructural WM changes were found to be widespread, interrupting structural pathways connecting remote brain regions and thus playing an important role in changes in cognitive function [13]. In early MS, a stronger structural-to-functional coupling was identified, suggesting that the ability of the brain to reorganise functional networks could diminish at later stages of the disease and could no longer compensate the MS-related structural damage [14,15]. Studies exploring treatment effects in GM for LUT symptoms in MS had proven difficult without the assessment of the degree of WM abnormalities between the relevant GM regions [16]. Therefore, it is important to investigate not only the changes in GM in MS but also the WM impairment. In patients with other WM disorders, namely small vessel disease, structural MRI studies suggest that the anterior thalamic radiation and the superior longitudinal fasciculus are specifically important for LUT functions and are involved in continence control [10,17], and WM hyperintensity (WMH) in the anterior corona radiata and the cingulate gyrus is reported to predict urinary incontinence and degree of bother [18].

Diffusion-weighted imaging (DWI) evaluates the microscopic random thermal motion of water, and is used to provide microstructural information on WM in neurological diseases [19,20,21,22]. Along WM tracts, water diffusion is more rapid, while it is slower in the direction perpendicular to the fibres [23]. This property is captured—to some extent—by diffusion tensor imaging (DTI), from which it is possible to obtain several scalar indices (fractional anisotropy, FA; mean diffusivity, MD; axial diffusivity, AD; radial diffusivity, RD) commonly used to characterise tissue microstructure properties [24]. FA is the most frequently used index in clinical studies for its better sensitivity to tissue microstructure property changes and has also been shown to be reduced in the WM of pwMS, compared to healthy people [25,26,27]. 

In this study, we aim to use DTI to explore the microstructural WM changes in a cohort of pwMS and compare differences in patients with and without LUT symptoms using voxel-wise analysis, and correlations between DTI measures (FA) and clinical scores of OAB, without a priori assumption on the location of the expected changes.

## 2. Materials and Methods

### 2.1. Subjects

Women with a diagnosis of MS according to the McDonald criteria [28], with and without LUT symptoms, attending the out-patient clinic at a tertiary-level teaching hospital, were included between September 2015 and August 2017. Individuals who were pregnant or breast feeding, having any contraindications for MRI, had a previous history of craniocerebral injuries or surgeries, had any other known neurological disorders, expercognitive impairment assessed by a Mini-Mental State Examination (MMSE < 23) [29], scored > 6.5 on the Expanded Disability Status Scale (EDSS) [30], reported an MS relapse in the previous 3 months, were undergoing treatment for LUT symptoms within the previous 6 months, had evidence for additional active urological disease or anatomical anomalies of the urogenital system during assessments, had undergone surgeries to the urogenital tract which could influence LUT symptoms, or had metabolic disease were excluded (see Appendix A). Considering that the 2017 revision of the McDonald criteria [31] was published during the study, all recruited MS patients were screened based on the 2017 McDonald criteria to make sure that the included MS patients fulfilled the new diagnostic criteria. Healthy controls (HCs) without diagnosed neurological disease or LUT symptoms were recruited. The study protocols were approved The Joint Medical Ethics Committee of the National Hospital for Neurology and Neurosurgery and the Institute of Neurology, London (protocol No. is 13/0523; REC reference is 14/LO/1636) and all subjects were fully informed and consented before participating.

### 2.2. Clinical Assessments

The neurological and uro-neurological examinations were performed for pwMS and HCs (JNP and AT). LUT symptoms were evaluated using a standard three-day bladder diary and a validated questionnaire, the Urinary Symptom Profile (USP) [32]. The USP questionnaire is a comprehensive evaluation of LUT symptoms that assesses three domains: OAB, stress urinary incontinence, and low stream [32]. Patients were classed as either having no LUT symptoms (MS-no-LUTS) or having OAB (MS-OAB) according to their history of LUT symptoms, the three-day bladder diary, and the USP questionnaire’s OAB sub-score. 

### 2.3. Imaging Acquisition

MRI scanning was performed using a 3.0 Tesla scanner (Philips Achieva, Philips Medical Systems, Best, The Netherlands). Proton density (PD) and T2-weighted images were acquired in the axial–oblique plane parallel to the anterior–posterior callosal line using the following parameters: Time Echo (TE)1/TE2 = 19/85 ms; Time Repetition (TR) = 3500 ms; Field Of View (FOV) 240 × 180 mm^2^; NEX = 1, voxel size = 1 × 1 × 3 mm^3^; 50 slices; and scan time = 4:01 min. DTI was also performed in the axial–oblique plane, with 32 distributed diffusion encoding directions (b = 0 and b = 1000 s/mm^2^) and the following parameters: TE = 92 ms; TR = 9714 ms; FOV 248 × 248 mm^2^; NEX = 1; voxel size = 2 × 2 × 2 mm^3^; 70 slices; and scan time = 6:46 min.

### 2.4. Lesion Masking

WM lesions were identified on both PD and T2-weighted images and manually segmented on PD images using Jim 6.0 (http://www.xinapse.com/Manual/, accessed on 1 March 2017). The lesion volume was then calculated and further used as a covariate in the statistical analysis.

### 2.5. MRI Image Processing and Tract-Based Spatial Statistics (TBSS) Analysis

Image processing and tract-based spatial statistics (TBSS) were performed using the FMRIB Software Library (FSL, Oxford, UK, https://fsl.fmrib.ox.ac.uk/fsl, accessed on 1 September 2017). The diffusion-weighted images were corrected for eddy current-induced distortions and movements using FSL [33]. DTI fitting and calculation of FA maps were performed using the NiftyFit toolbox [34].

The standard TBSS pipeline was applied to the data: all FA images were non-linearly registered [35] to the FMRIB58_FA template in MNI152 space (MNI, McConnell Brain Imaging Centre), and then skeletonised and concatenated into a 4D file (skeletonized). The WM skeleton was created with an FA threshold of 0.2. The JHU ICBM-DTI-81 White-Matter atlas and the JHU White-Matter Tractography atlas were used to identify the regions of interest emerging from the statistical analysis of the skeletonised FA of pwMS subgroups and HCs [36,37].

### 2.6. Statistical Analysis

Descriptive statistics of age, EDSS, lesion volume, disease duration, and the OAB sub-score from the USP questionnaire (USP-OAB) were expressed as means and standard deviations and all variables were checked for skewness and presence of outliers. SPSS version 24 was used for the statistical analysis; a *p* value < 0.05 was considered statistically significant, and a *p* value < 0.1 was considered a favourable statistical trend.

The design matrix and contrast files were created by the General Linear Model (GLM) tool (https://fsl.fmrib.ox.ac.uk/fsl/fslwiki/GLM, accessed on 1 December 2017) and passed to the Randomise tool (https://fsl.fmrib.ox.ac.uk/fsl/fslwiki/Randomise, accessed on 1 December 2017) for nonparametric permutation inference (5000 permutations). The Threshold-Free Cluster Enhancement (TFCE) option was adopted for all tests in Randomise (*p* < 0.05 as statistical significant; *p* < 0.1 as statistical trend) [38].

Differences between the pwMS and HCs were analysed for internal validation, adjusting for age. Comparisons between the two pwMS sub-groups (MS-no-LUTS and MS-OAB) were performed to identify WM changes potentially responsible for LUT symptoms in MS, adjusting for age and EDSS. Correlation analysis (Spearman correlation coefficient, 2-tailed) was performed to explore any associations between diffusion measures (FA) and clinical scores of OAB (USP-OAB) in pwMS. All regressors added to the regression model were mean-centred across all subjects.

### 2.7. Bullseye Representation

Differences in the distribution and frequency of WM changes were plotted in a bullseye plot of WM regions [39]. The regional representation was based on the division of the WM and deep GM volume into sectors (representing the lobes) and radial regions (representing the distance from the ventricles) [39]. Aggregation of cortical regions obtained from the Geodesic Information Flow (GIF) parcellation algorithm [40] applied to the brain in MNI space was used to obtain cortical lobes (or deep GM regions), and WM voxels were allocated to the closest cortical lobe. The lobes were illustrated as FRONT (frontal lobe), PAR (parietal lobe), TEMP (temporal lobe), OCC (occipital lobe), and BGIT (the basal ganglia, thalami, and infratentorial regions from both sides) [39]. Radially, the application of the Laplace equation between the ventricular and cortical surfaces enabled the creation of a normalised distance measure that was divided into four equal parts providing the four equidistant radial layers [39]. Overall, this systematic division of the volume of interest resulted in thirty-six regions (four layers and nine lobar separations) [39].

This regional division of the WM volume allows for a local representation of the whole-brain results. In this study, distribution plots reflect the ratio between the number of voxels of interest (significant values) located in the specific region and the overall number of significant voxels, while the frequency plots were drawn as the ratio between number of significant voxels in a given region and volume of this region. The lesion volume frequency plots represent the ratio between the median and the interquartile range (IQR, measuring statistical dispersion) of lesion volume in the given region and the volume of that region.

## 3. Results

### 3.1. Subsection

#### 3.1.1. Participants

Thirty right-handed women pwMS and fourteen women HCs fulfilling the recruitment/inclusion criteria participated in this study. Three pwMS reporting predominantly voiding problems were excluded from the data analysis between MS-no-LUTS and MS-OAB, and one patient was excluded after a subsequent change in diagnosis. Demographic characteristics are presented in Table 1. 

#### 3.1.2. Group Difference Analysis

**Figure 1:**
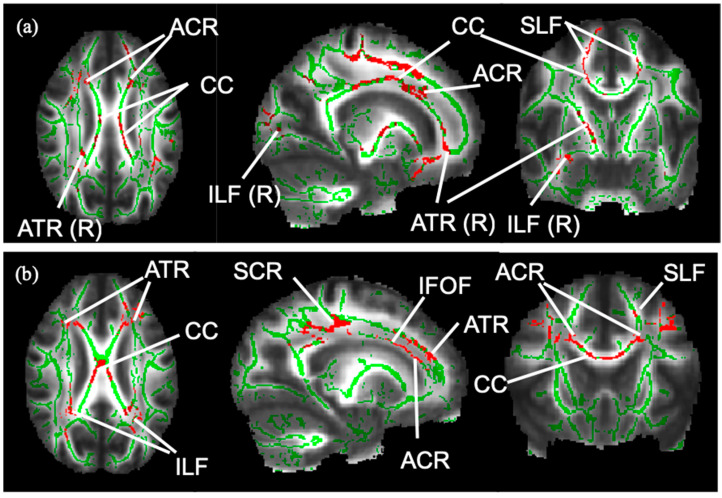
WM skeleton and significant results from TBSS. The WM skeleton in green is created based on voxels indicative of WM across pwMS (n = 26). (**a**): Voxels in red are indicative of a significant FA reduction in MS-OAB compared to MS-no-LUTS, adjusting for age and EDSS (*p* = 0.072). (**b**): Voxels in red show significant negative correlation between FA and USP-OAB sub-score (*p* = 0.021) in the following tracts: ACR: anterior corona radiata; ATR: anterior thalamic radiation; CC: corpus callosum; IFOF: inferior fronto-occipital fasciculus; ILF: inferior longitudinal fasciculus; R: right-sided; SCR: superior corona radiata; SLF: superior longitudinal fasciculus.

**Figure 2:**
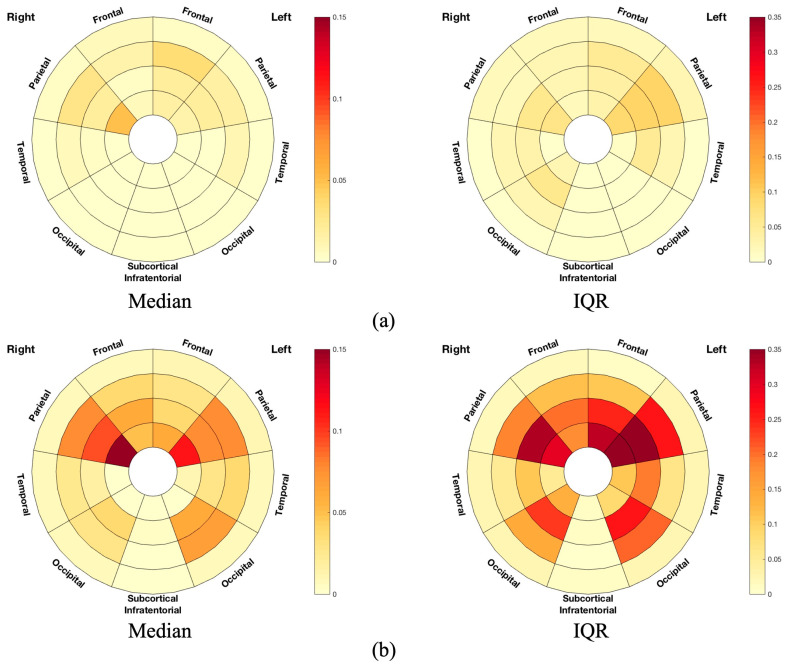
Frequency plots of lesion volume in terms of median and IQR in (**a**) MS-no-LUTS and (**b**) MS-OAB. The frequency plots were drawn as the ratio between number of significant voxels in a given region and volume of this region. The plots are considered radially between the ventricles and the cortical grey matter discretized into four equidistant layers, derived from the solution to the Laplace equation [41]. The colour bars from bottom to top indicate the lesion volume from lowest to highest. IQR: interquartile range. Frontal: frontal lobe; Occipital: occipital lobe; Parietal: parietal lobe; Subcortical Infratentorial: the basal ganglia, thalami, and infratentorial regions from both sides; Temporal: temporal lobe.

Differences in the FA of the WM skeleton between the three groups were statistically significant (Table 2). The WM skeleton and significant results of group difference from TBSS are illustrated in the images in Figure 1. Compared with the HC group, FA was significantly reduced in the MS group after adjusting for age, in voxels, across whole-brain WM regions, especially the corpus callosum. Compared with MS-no-LUTS, there was a significant FA reduction in the MS-OAB group after adjusting for EDSS (*p* = 0.047), and a trend for significance after adjusting for age and EDSS (*p* = 0.072) (Table 2). A greater lesion volume was seen in the MS-OAB group (Figure 2).

#### 3.1.3. Correlation Analysis 

Table 3 illustrates the results of the correlation analysis and relative significance. When all the participants were analysed as a single group (n = 43), negative correlations were observed between FA and age (*p* = 0.018) and USP-OAB sub-score (*p* = 0.001). In the pwMS cohort (n = 26), significant negative correlations were observed between FA and age (*p* = 0.008) and the USP-OAB sub-score (*p* = 0.021). 

#### 3.1.4. WM Tract Analysis Using TBSS

Voxels in the corpus callosum, anterior corona radiata bilaterally, right anterior thalamic radiation, superior longitudinal fasciculus bilaterally, and right inferior longitudinal fasciculus showed a trend for significance in group difference in FA between MS-no-LUTS and MS-OAB, after adjusting for age and EDSS score (Figure 1a). A significant correlation was seen between decreasing FA and increasing USP-OAB sub-score in voxels in the corpus callosum, anterior corona radiata bilaterally, superior corona radiata bilaterally, superior longitudinal fasciculus bilaterally, inferior longitudinal fasciculus bilaterally, and inferior fronto-occipital fasciculus bilaterally (Figure 1b).

**Figure 3:**
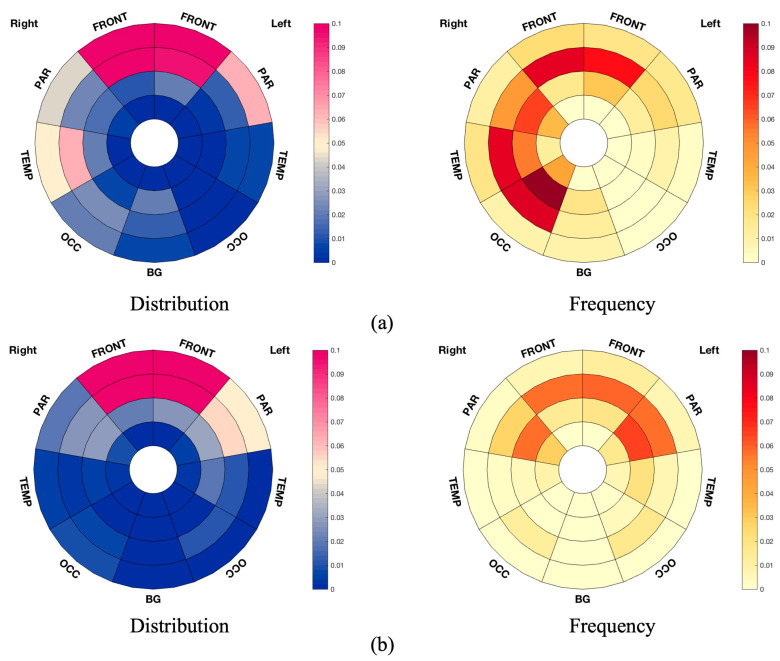
Bullseye plots showing significant results from TBSS. (**a**) Bullseye plots showing reduced FA in MS-OAB, compared with MS-no-LUTS, adjusting for age and EDSS (*p* = 0.072). (**b**) Bullseye plots showing negative correlation between FA and USP-OAB sub-score across pwMS (n = 26, *p* = 0.021). The distribution plots reflect the ratio between the number of voxels of interest (significant values) located in the specific region and the overall number of significant voxels, while the frequency plots are drawn as the ratio between number of significant voxels in a given region and volume of this region. The plots are considered radially between the ventricles and the cortical grey matter discretized into four equidistant layers, derived from the solution to the Laplace equation [41]. The colour bars from bottom to top indicate the number of voxels from lowest to highest at the significance level. FRONT: frontal lobe; BG: the basal ganglia, thalami, and infratentorial regions from both sides; OCC: occipital lobe; PAR: parietal lobe; TEMP: temporal lobe.

Bullseye plots show the lobar involvement of group difference and correlation analysis, respectively (Figure 3).

## 4. Discussion

Current understanding of the central neural network controlling LUT functions is centred around different GM cortical and subcortical regions such as the thalamus, insula, anterior cingulate cortex, mPFC, and lPFC [8]. Findings in the neural network that correlate with LUT dysfunction have been demonstrated in pwMS. In MS, the WM lesion distribution correlates with the clinical manifestations and disease course [42]. The role of the connecting WM tracts in the regulation of LUT functions remains speculative [8] and this study aimed to explore the microstructural WM changes in association with OAB symptoms in pwMS. In this study, we explored differences in WM microstructure in MS-OAB and MS-no-LUTS by evaluating changes in FA using diffusion-weighted MRI. We identified FA reduction in the pwMS cohort compared with HC, which was consistent with the previous literature [25,43]. There were two salient findings that demonstrate the relationship between changes in WM tracts and the occurrence of OAB symptoms. 

Reductions in FA were observed in the MS-OAB group compared with MS-no-LUTS (Table 2) in regions including the corpus callosum, anterior corona radiata, anterior thalamic radiation, superior longitudinal fasciculus, and inferior longitudinal fasciculus, and the reductions in FA were consistent with previous studies [17,18]. These tracts were particularly susceptible in pwMS and correlated with LUT symptoms. Among the identified tracts, WM changes in the corpus callosum have been widely reported in pwMS, accounting for various LUT functions; WM changes in the anterior thalamic radiation, superior longitudinal fasciculus, and inferior longitudinal fasciculus had also been reported in pwMS, accounting for cognitive functions [25]. After controlling for age and EDSS, taken as a measure of overall motor and functional disability, a significant FA reduction was seen in WM tracts in MS-OAB, suggesting that involvement of these WM tracts results in LUT symptoms. Taking into consideration the working model of LUT control based on previous studies evaluating GM structures [8], the findings from the present study suggested the presence of a suprapontine WM circuit supporting the LUT functional network, connecting the different GM regions involving the thalamus, insula, anterior cingulate cortex, and prefrontal cortex. In addition, WM studies assessing WMH in a cohort of elderly community-dwelling women from Kuchel et al. and Tadic et al. speculated the involvement of the cingulum, anterior corona radiata, anterior thalamic radiation, superior longitudinal fasciculus, and superior frontal-occipital fasciculus for urinary incontinence [17,18]. Our study confirmed the presence of FA reductions in these specific WM tracts as well using an established quantitative MRI technique and statistical analysis of the whole WM skeleton.

WM changes were particularly observed in the frontal WM as expected. Bladder function, including attention and information processing speed in integrating the awareness of bladder filling and voiding, was associated with cognitive performance, pointing out a strong overlap of the brain structures involved [44]. Regions in frontal WM were demonstrated to correlate with information processing speed and attention [15]. The role of the prefrontal lobe in continence was established through seminal work by Andrew and Nathan [45], and the orbitofrontal or lPFC has been demonstrated to have extensive connections with the limbic system, including the insula, which was found to be the homeostatic afferent area projecting the sensation of bladder filling [46,47], and the anterior cingulate cortex, which mediated LUT functions together with the supplementary motor area through the perception of the sensation of urgency and resultant contraction of the urethral sphincter [48]. Moreover, differences were seen to extend beyond the frontal WM, and rather unexpectedly greater WM changes were seen in the entire non-dominant (right) hemisphere in the MS-OAB group (Figure 3a). Previous studies evaluating the cerebral control network of LUT functions had similarly demonstrated BOLD signal changes preferentially affecting the non-dominant insula and right lPFC [8]. There was a non-dominant hemisphere prevalence presented in the median lesion volume plot (Figure 2b), and lesions of the right hemisphere would be consistent with the expected findings in the literature [18]. Previous studies demonstrated a correlation between interoceptive awareness and right GM regions, including the right insula and right thalamus [49,50,51], and the findings from this study suggest that WM changes in the right hemisphere can also result in changes in visceral functions manifesting with LUT symptoms. 

A negative correlation was observed between FA and the USP-OAB sub-score, suggesting that a lower FA was associated with more severe OAB symptoms (Table 3). Considering that an important role for WM changes in the anterior corona radiata was shown to predict urinary incontinence severity in elderly women with small vessel disease [10,18], this study confirmed the importance of this tract in the neural control of LUT functions; moreover, our results suggested that there were additional WM tracts that, when affected by microstructure alterations, could contribute to LUT dysfunction (OAB) in neurological diseases (MS). It would be pertinent for future studies to confirm these findings in terms of the correlation with specific symptoms of OAB, such as urgency and frequency, and in other neurological disorders, such as small vessel disease.

There are some limitations of this study. In the group difference analysis, only a trend towards a significantly reduced FA (*p* = 0.072) was observed in MS-OAB compared with MS-no-LUTS, after adjusting for age and EDSS. The small sample size and the differences in group size were likely to have been factors affecting the group difference results, though LUT symptoms are reported in 80% of pwMS and therefore a cohort of pwMS without LUT symptoms is uncommon and difficult to recruit. Secondly, LUT symptoms presented heterogeneously in MS, and the cohort we investigated was only a subset of a more complex group. There could have been a selection bias to select only female patients (EDSS ≤ 6.5) in our study, but this could be interpreted as an intended benefit of having a more uniform study population. Additionally, only the suprapontine subcortical WM changes were evaluated in this study and LUT symptoms can also arise from spinal cord lesions [1], which were not assessed. Lastly, the EDSS score used for measuring MS disability includes a bladder functional symptom score which may account for a decrease in significance when regressing EDSS for LUT symptoms. Greater correlation with involvement of WM in the non-dominant hemisphere needs to be evaluated in further studies to understand whether this contributes to the mechanisms of LUT dysfunction only in MS or whether it is a salient characteristic in LUT dysfunction.

## 5. Conclusions

The findings of this study demonstrate an association between more severe OAB symptoms and more microstructural WM abnormalities in both frontal lobes and the rest of the non-dominant hemisphere in pwMS, using diffusion MRI. WM lesions affecting specific WM tracts, including the corpus callosum, anterior corona radiata bilaterally, superior corona radiata bilaterally, superior longitudinal fasciculus bilaterally, inferior longitudinal fasciculus bilaterally, and inferior fronto-occipital fasciculus bilaterally, can result in more severe OAB symptoms. Our findings suggest that the WM tracts play an important role in the neural control of LUT functions.

## Figures and Tables

**Table 1:** Demographic characteristics of healthy controls and pwMS.

	HCs	pwMS	MS-no-LUTS	MS-OAB	*p*-Value	
n	14	29	9	17	N/A ^a^	N/A ^b^
Mean age, years (SD)	48.5 (20.0)	43.3 (9.4)	37.5 (8.9)	46.1 (8.6)	0.641 ^a^	0.026 ^b,^*
Mean EDSS (SD)	N/A	2.3 (1.8)	0.9 (0.9)	2.9 (2.0)	N/A ^a^	0.004 ^b,^**
Median lesion volume, ×10^3^ mL (range)	N/A	7.7 (0.2–69.6)	1.9 (0.2–13.7)	8.8 (1.0–69.6)	N/A ^a^	0.055 ^b,^*
Median disease duration, years (range)	N/A	10.0 (0.8–42.0)	4.0 (1.0–14.0)	12.3 (0.8–42.0)	N/A ^a^	0.029 ^b,^*
Mean USP-OAB (SD)	1.1 (1.7)	5.9 (4.7)	1.0 (1.0)	8.8 (3.7)	<0.001 ^a,^**	<0.001 ^b,^**

EDSS: Expanded Disability Status Scale; HCs: healthy controls; MS: multiple sclerosis; MS-no-LUTS: MS patients without lower urinary tract symptoms; MS-OAB: MS patients with OAB; N/A: not applicable; OAB: overactive bladder; pwMS: patients with MS; USP: Urinary Symptom Profile; USP-OAB: USP OAB sub-score. Statistically significant difference was found between HCs and pwMS. MS-no-LUTS and MS-OAB were tested using Chi Square test, student’s *t*-test, and Mann–Whitney U test. ^a^: statistical difference between HCs and pwMS; ^b^: statistical difference between MS-no-LUTS and MS-OAB. *: *p* < 0.05; **: *p* < 0.01.

**Table 2:** FA values of mean skeleton in HCs, pwMS, and pwMS sub-groups.

	FA of Mean Skeleton (Median, Range)	*p*-Value (Adjusted for Age)	*p*-Value (Adjusted for EDSS)	*p*-Value (Adjusted for Age + EDSS)
HCs (n = 14)	0.4590268 (0.4404533, 0.4853403)	0.004 **	N/A	N/A
pwMS (n = 29)	0.4338049 (0.3303200, 0.4874299)			
MS-no-LUTS (n = 9)	0.4446728 (0.4288627, 0.4740112)	0.159	0.047 *	0.072
MS-OAB (n = 17)	0.4299469 (0.3305743, 0.4859033)			

FA: fractional anisotropy; HCs: healthy controls; MS: multiple sclerosis; MS-no-LUTS: MS patients without lower urinary tract symptoms; MS-OAB: MS patients with OAB; N/A: not applicable; pwMS: patients with MS. Statistically significant difference was found between HCs and pwMS. MS-no-LUTS and MS-OAB were tested using Mann–Whitney U test. *: *p* < 0.05; **: *p* < 0.01.

**Table 3:** Correlations between FA values and clinical scores.

	Age	EDSS	USP-OAB
FA of mean skeleton of all subjects (n = 43)	r = −0.36 * p = 0.018	N/A	r = −0.493 ** p = 0.001
FA of mean skeleton of pwMS (n = 26)	r = −0.51 ** p = 0.008	r = −0.271 *p* = 0.181	r = −0.449 * p = 0.021

Spearman’s correlations between FA value and age; EDSS and USP-OAB sub-score were tested in HCs and pwMS. EDSS: Expanded Disability Status Scale; FA: fractional anisotropy; MS: multiple sclerosis; N/A: not applicable; *p*: *p*-value of correlation; pwMS: patients with MS; r: Spearman’s correlation coefficient (two-tailed); USP-OAB: Urinary Symptom Profile OAB sub-score. *: *p* < 0.05; **: *p* < 0.01.

## Data Availability

The data presented in this study are available on request from the corresponding author. Data are not publicly available due to ethical reasons.

## Appendix A. Summary of Inclusion and Exclusion Criteria of This Study

**Inclusion and Exclusion Criteria**
Inclusion Criteria - At least 18 years old - Able to understand the patient information sheet - Written informed consent to take part and follow the requirements of the protocol For patients with MS: - Diagnosis according to the McDonald criteria - Having a score on the Kurtzke Expanded Disability Status Scale ≤ 6.5 - Free of relapses in the previous 3 months For patients with idiopathic LUT symptoms: - Routine clinical assessments excluding a neurological cause for symptoms For patients with predominant storage symptoms: - Urinary urgency (≥2 episodes per week), urinary frequency (>8/24 h), and/or incontinence - Post-void residual volume < 150 mL For patients with predominant voiding symptoms: - Hesitancy, slow or intermittent stream, straining to void, or relying on catheterisation for bladder emptying For patients with mixed storage and voiding LUT symptoms: - Urgency (≥2 episodes per week), urinary frequency (>8/24 h), and/or incontinence - Hesitancy, slow or intermittent stream, straining to void, or relying on catheterisation for bladder emptying For patients with neurological disease without LUT symptoms: - No episodes of urinary urgency or incontinence; urinary frequency < 8/24 h - No voiding symptoms; post-void residual volume < 150 mL For healthy subjects: - Good mental and physical health - Normal functioning of the LUT - No episodes of urinary urgency or incontinence; urinary frequency < 8/24 h - No malignancy or previous surgery of the LUT or genitalia - No previous spine or pelvic surgery
Exclusion Criteria - Pregnant, breast feeding, or planning to become pregnant during the study duration - Any contraindications to having MRI (e.g., ferromagnetic implants), as assessed by the NMR unit’s MRI safety checklist - Craniocerebral injuries or surgeries - Known neurological disease other than that being used for the investigation - Cognitive impairment, as assessed by a Mini-mental State Examination (MMSE) score < 23 - Presence of additional active urological disease that might explain LUT symptoms - Surgery of the LUT or genitalia within the last year or that is related to the LUT symptoms, as per the discretion of the investigator - Any anatomical anomaly of LUT/genitalia - Active LUT malignancy or metabolic disease, as per the discretion of the investigator - For the functional brain imaging part, additional exclusion criteria apply: - Receiving concomitant treatment for LUTS - Received tibial nerve stimulation or intravesical Botulinum A toxin injections within the previous six months - If on an antimuscarinic medication, unwilling to discontinue for at least 5 days prior to having MRI assessment
